# Epigenetic modifications in pancreas development, diabetes, and therapeutics

**DOI:** 10.1002/med.21878

**Published:** 2022-01-04

**Authors:** Suneesh Kaimala, Challagandla Anil Kumar, Mohammed Z. Allouh, Suraiya Anjum Ansari, Bright Starling Emerald

**Affiliations:** ^1^ Department of Anatomy, College of Medicine and Health Sciences United Arab Emirates University Al Ain Abu Dhabi UAE; ^2^ Department of Biochemistry College of Medicine and Health Sciences, United Arab Emirates University Al Ain Abu Dhabi UAE; ^3^ Zayed Center for Health Sciences United Arab Emirates University Al Ain Abu Dhabi UAE

**Keywords:** DNA methylation, epigenetic modulators, gestational diabetes, histone modifications, pancreas, type 1 diabetes, type 2 diabetes

## Abstract

A recent International Diabetes Federation report suggests that more than 463 million people between 20 and 79 years have diabetes. Of the 20 million women affected by hyperglycemia during pregnancy, 84% have gestational diabetes. In addition, more than 1.1 million children or adolescents are affected by type 1 diabetes. Factors contributing to the increase in diabetes prevalence are complex and include contributions from genetic, environmental, and epigenetic factors. However, molecular regulatory mechanisms influencing the progression of an individual towards increased susceptibility to metabolic diseases such as diabetes are not fully understood. Recent studies suggest that the pathogenesis of diabetes involves epigenetic changes, resulting in a persistently dysregulated metabolic phenotype. This review summarizes the role of epigenetic mechanisms, mainly DNA methylation and histone modifications, in the development of the pancreas, their contribution to the development of diabetes, and the potential employment of epigenetic modulators in diabetes treatment.

**Abbreviations**
ASH: absent small and homeotic disks protein 1 homolog
BET: bromodomain extra‐terminal protein
CARM1: coactivator‐associated arginine methyltransferase 1
CML: chronic myeloid leukaemia
Cpa1: carboxypeptidase A1
CREB: camp response element‐binding
*CTLA4*
: cytotoxic T‐lymphocyte‐associated protein 4
DMRs: differentially methylated regions
DNMT: DNA methyltransferase
DOT1L: DOT1 like histone lysine methyltransferase
EHMT2: euchromatic histone lysine methyltransferase 2
EHMT2: euchromatic histone lysine methyltransferase 2
Enpp1: ectonucleotide pyrophosphatase/phosphodiesterase 1
ER: endoplasmic reticulum
EZH2: enhancer of Zeste 2 Polycomb Repressive Complex 2 Subunit
FOXA2: forkhead box protein A2
GABPA: calmodulin kinase II GA binding protein transcription factor subunit alpha
GATA4: GATA binding protein 4
GCG: glucagon
GDM: gestational diabetes mellitus
GLP1: glucagon‐like peptide
GO: gene ontology
GWAS: genome‐wide association studies
G‐CSF: granulocyte colony‐stimulating factor
Hdacs: histone deacetylases
HDMS: histone demethylases
HFD: high‐fat diet
HGP: hepatic glucose production
HLA: human leukocyte antigen
HLA: human leukocyte antigen
HMTs: histone methyltransferases
IDDM: insulin‐dependent diabetes mellitus
IFN‐gamma: interferon gamma
IL2RB: interleukin‐2 receptor β
IL6: interleukin‐6
Ins: insulin
IUGR: intrauterine growth retardation model
Jmjd3: jumonji domain‐containing protein 3
Kdaci: histone lysine deacetylase inhibitors
KH2: hexokinase 2 HK2
LBW: low birth weight
LDLc: low‐density lipoprotein c
LINE: long interspersed nuclear elements
LPL: lipoprotein lipase
MAFB: MAF bzip transcription factor B
MeDIP: methylated DNA immunoprecipitation
MetS: metabolic syndrome
MLL1‐5: mixed‐lineage leukaemia 1‐5
MSK‐1/2: mitogen and stress‐activated kinase 1/2 Embryonic day 12.5 E12.5
MVP: multivariate positions
Nab: sodium butyrate
Nab: sodium butyrate
NAFLD: nonalcoholic fatty liver disease
Neurog3: neurogenin 3
NNAT: neuronatin
NOD: nonobese diabetic
NSD1: nuclear receptor binding SET domain protein 1
Pcldx1: phosphatidylinositol specific phospholipase c x domain containing 1
PCNA: proliferating cell nuclear antigen
PDX1: duodenal homeobox 1
Prmts: protein arginine methyltransferases
RIZ1: retinoblastoma protein‐binding zinc finger protein 1
SAHA: suberoylanilide hydroxamic acid
SET1A: SET domain containing 1A
SET1B: SET domain containing 1B
SET2: SET domain containing 2
SET7: enhancer‐of‐zeste, trithorax domain‐containing lysine methyltransferase 7
SHH: sonic hedgehog
SIRT1: sirtuin 1
Slc27a2: solute carrier family 27 member 2
SNP: single‐nucleotide polymorphism
SOX9: SRY‐box transcription factor 9
SREBF2: sterol regulatory element‐binding transcription factor 2
STZ: streptozotocin
STZ: streptozotocin
SUV39H1: suppressor of variegation 3‐9 homolog 1
SUV39H2: suppressor of variegation 3‐9 homolog 2
T1D: type 1 diabetes
T2D: type 2 diabetes
TLR: toll‐like receptor
TNFALPHA: tumor necrosis factor
TSA: trichostatin A
Txnip: thioredoxin interacting protein
UHRF1: ubiquitin‐like with PHD and ring finger domains 1
VPA: valproic acid
Wpc: weeks postconception

## 1 INTRODUCTION

Metabolism is a broad term used to describe a set of life‐sustaining chemical reactions occurring in the body. Metabolism is a tightly controlled process involving different systems such as the nervous, digestive, and endocrine systems. Any errors in metabolism would cause an imbalance in the functional homeostasis of the organism and result in diseases collectively called metabolic syndrome (MetS) that include insulin resistance, hyperglycemia, cardiovascular diseases, hyperinsulinemia, obesity, hyperlipidemia, and hypertension.^1^

Diabetes mellitus is a disease caused by abnormal glucose metabolism, resulting in consistently high blood glucose levels. Insufficient insulin levels caused by reduced insulin secretion from the pancreas lead to Type 1 diabetes (T1D), whereas defective insulin signalling causing inefficient utilization of glucose by muscles leads to Type 2 diabetes (T2D). Gestational diabetes mellitus (GDM) is a kind of diabetes that develops during pregnancy. Diabetes affects the nervous system, cardiovascular system, kidneys, eyes, bones, and immune systems and is caused by the combination of genetic, environmental, and epigenetic factors.^2^ In this review, we summarize the epigenetic factors, mainly focusing on DNA methylation and histone modifications that regulate the development of the pancreas and changes in them that lead to diabetes. We have also included the role of epigenetic modulators in diabetes diagnosis and treatment.

## 2 HISTONE MODIFICATIONS

Histones are basic globular proteins that aid in packaging long DNA in an orderly manner within the nucleus. This, in turn, is responsible for the regulation of gene expression within the cell. The nucleosome structural unit consists of approximately 146 base pairs of DNA wrapped around an octamer that is made up of two copies of each of the four core histone proteins (H2A, H2B, H3, and H4) in a left‐handed super‐helical turn.^3^, ^4^ Histone H1 acts as a linker of the nucleosomes and contributes to the binding of the DNA to the nucleosome, and contributes to the structural integrity of chromatin, mainly through the modulation of protein phosphorylation and O‐glycosylation.^5^, ^6^ Histones have unstructured N‐terminal tails that undergo different posttranslational modifications, such as methylation (arginine and lysine), acetylation (lysine), ubiquitination (lysine), sumoylation (lysine), phosphorylation (serine and threonine), ADP‐ribosylation, and glycosylation, which alter the functional properties and structures of chromatin.^7^

Histone methyltransferases (HMTs) are enzymes that catalyze histone methylation by transferring one to three methyl groups from S‐adenosyl methionine to the lysine or arginine residues of the histone tails. Histone lysine methyltransferases include histone H3K4 methyltransferases such as mixed‐lineage leukemia 1‐5 (MLL1‐5), SET domain‐containing 1A, 1B (SET1A, SET1B) and Absent small and homeotic disks protein 1 homolog (ASH), histone H3K9 methyltransferases such as euchromatic histone lysine methyltransferase 2 (EHMT2), Suppressor of variegation 3‐9 homolog 1, 2 (SUV39H1, SUV39H2), and retinoblastoma protein‐binding zinc finger protein 1 (RIZ1), histone H3K36 methyltransferases SET domain containing 2 (SET2), and nuclear receptor binding set domain protein 1 (NSD1), H3K79 methyltransferase, DOT1 like histone lysine methyltransferase (DOT1L), enhancer of zeste 2 polycomb repressive complex 2 subunit (EZH2) which methylates H3K27. Histone methylation is generally associated with transcriptional repression (H3K9me, H3K27me), although methylation of some lysine's (e.g., H3K4, H3K79) and arginine residues on H3 and H4 are shown to be associated with transcriptional activation.^8^ Histone methylation is a reversible process catalyzed by histone demethylases (HDMs);^9^ for example, lysine‐specific demethylase 1 (a flavin‐dependent monoamine oxidase) demethylates the mono‐ and di‐methylated lysine residues H3K4 and H3K9.^10^

The acetylation of histones generally influences gene expression through the activation of transcription. The acetylation of lysine residues catalyzed by histone acetyltransferases neutralizes the positive charge on histones. This, in turn, decreases the interaction of the N‐termini of histones with negatively charged DNA phosphate groups. This changed tail transforms compact chromatin into an open structure and gives access to transcriptional machinery, which results in gene transcription.^11^, ^12^ Histone deacetylases (HDACs) catalyze the removal of acetyl groups from histone tails to coenzyme A.^13^ Based on their intracellular localization, HDACs are classified into three classes: Class I—HDAC 1, 2, 3, and 8 (exclusively localized to the nucleus); Class IIa and b—HDAC 4, 5, 6, 7, 9, and 10 (predominantly residing in the cytoplasm and shuttling in and out of the nucleus); and Class III—sirtuin enzymes (SIRT) 1, 2, 3, 4, 5, 6, and 7 (localized in either the nucleus, cytoplasm, or mitochondria).^14^, ^15^ Histone modifications in multicellular organisms are also heritable, as seen with H3K27 and H3K4 modifications during mitosis, and are catalyzed by the polycomb‐group (PcG) and trithorax‐group (TrxG) genes.^16^

The phosphorylation of histones by ATP‐dependent protein kinases also plays a role in regulating histone biological activity, which mainly occurs during mitosis and meiosis.^17^ This regulation, which is coordinated both in space and time, plays a critical role in the modulation of the nucleosome structure and, thus, DNA accessibility.^18^ The phosphorylation of H2A (serine S1 and threonine T119), H3 (serine S10 and S28, and threonine T3 and T11), and H4 (serine S1) results in transcriptional activation and promotes mitosis and meiosis.^19^ H2A phosphorylation involves DNA repair, whereas H4 phosphorylation promotes spermatogenesis. H1 phosphorylation regulates the decondensation of chromatin and activation of transcription, and the phosphorylation of H3 is critical for cytokine‐induced gene expression.^17^

The monoubiquitination of histones H2A and H2B had been shown to play critical roles in DNA repair, gene transcription, and messenger RNA (mRNA) translation.^19^ Ubiquitination results from the conjugation of a 76‐amino acid polypeptide to histones in a series of reactions involving three enzymatic steps.^20^, ^21^ H2B monoubiquitination is also a prerequisite for H3K4 and H3K79 methylation, whereas H2A K119 monoubiquitination is associated with transcriptional silencing facilitated through polycomb repressive protein complex 1 (PRC1) and PRC2 (EZH2) recruitment, which results in H3K27me3.^20^, ^21^, ^22^ It is important to recognize that it is the sum of different histone modifications and the DNA methylation profile of a specific cell type or tissue that specifies the outcome instead of a single modification.

### 2.1 Histone modifications during pancreas development

Pancreas development begins at Carnegie stage 10 (CS10) in humans, with the pancreatic bud developing from the foregut region of the endoderm, which is marked by diminishing levels of sonic hedgehog and increasing levels of pancreatic and duodenal homeobox 1 (PDX1).^23^ At CS13, microluminar structures for the drainage of acinar secretions into the intestine begin to develop. From CS13 to CS19, multipotent pancreatic progenitors develop into bipotent progenitors that could develop into pancreas or liver lineage and undergo extensive proliferation. Within this prospective liver domain, enhancer of zeste homolog 2 (EZH2), a component of PRC2, represses *PDX1* expression to restrain the induction of the ventral pancreas and allow liver specification.^24^ During the development of the pancreas in the pancreatic domain, p300‐dependent histone acetylation is reduced, which results in diminished expression of liver markers and activation of pancreatic markers leading to blocking of liver specification and specifying the pancreatic domain.^24^ Thus, the endoderm is prepatterned to specify the ventral pancreatic lineage by default, and liver specification is actively promoted by epigenetic regulation. In embryonic pancreas explants and in experiments using human embryonic stem cell cultures, inhibiting the expression of EZH2 results in increased pancreatic endocrine progenitors in vitro.^25^ Loss of EZH2 did not affect the dorsal pancreatic bud invagination and growth, suggesting that the dorsal pancreatic bud is specified independently of EZH2.^25^ However, increased specification of endocrine progenitors and β‐cells was observed if EZH2 was deleted at the pancreas progenitor stage. A basic helix‐loop‐helix transcription factor, Neurogenin 3 (Neurog 3), functions as a key regulator of cell fate specification in the developing pancreas. Dynamic changes in the expression of Neurog 3 in the progenitor cells regulate the H3K27 trimethylation (H3K27me3) at the promoter regions of key transcription factors involved in the transition of endoderm to pancreatic progenitor cells. During the development of the pancreas, genes that lose the H3K27me3 mark are generally transcriptional regulators, and they favour proendocrine fates, whereas genes that acquire the H3K27me3 mark are those that regulate cell division and morphogenesis. Deletion of jumonji domain‐containing protein 3 (*Jmjd3*), an H3K27me3 demethylase, results in impaired differentiation of progenitor cells to endocrine cell transition and islet formation.^26^ Bromodomain extra‐terminal protein (BET), a known regulator of pancreatic development, when inhibited, increased neurogenin 3 (Neurog3) expressing pancreatic progenitors, but reduced Insulin 1 (*Ins1*) expression in mouse embryonic pancreas explants. BETs, especially BRD4, is known to modulate RNA polymerase II (RNA pol II) activity by its interaction with the transcription initiation complex or by releasing a paused RNA pol II. The reduced expression of Ins1 in inhibitor‐treated cells could be because of the diminished RNA pol II modulatory effects of BETs. It is also possible that increases in Ngn3 in the pancreatic primordium cause premature differentiation into endocrine cells at the expense of progenitors, reducing the pool of cells available to become endocrine and exocrine cells. Thus, pancreata from inhibitor‐treated explants may harbour higher percentages of endocrine cells but lower numbers, accounting for reduced expression of insulin. Acinar cells were also increased by the BET inhibition, as indicated by the increase in the cells with the expression of carboxypeptidase A1 (Cpa1) and CelA markers.^27^ By CS19, the different cell lineages in the pancreas become clear with GATA binding protein 4 (GATA4) expressing peripheral cluster cells (tips), and GATA4 diminished central duct‐like structures (trunks). These peripheral tip cells are proacinar cells.^28^

In humans, NEUROG3 increases rapidly in the trunk cells during β‐cell mass formation. This is followed by the vascularization of β‐cell mass by 10‐week postconception (wpc) and the disappearance of SRY‐box transcription factor 9 (SOX9) in the trunk cells. By 12–13 wpc, islets become fully developed with α‐, β‐, γ‐, δ‐ and ε‐ cells.^29^ HDACs are required for the establishment of appropriate ratios of β‐ and δ‐cells. Rodent models with the loss‐ and gain‐of‐function of *Hdacs* have shown that the loss of *Hdac5* and *Hdac9* had an increase in β‐cell mass, and the mice lacking *Hdac4* and *Hdac9* had more δ‐cells.^30^ Studies using Xenopus embryos showed that the set domain‐containing protein 1 (Setd1), a HMT, plays a central role in specifying pancreatic cell identity. Developmentally, the transcription of*Setd7* is observed in endodermal cells before *Pdx1* transcription and leads to changes in specific epigenetic marks in chromatin, leading to pancreatic cell‐specific differentiation.^31^ The reduced expression of mitogen and stress‐activated kinase 1/2 (Msk 1/2) at embryonic Day 12.5 (E12.5) causes decreased phosphorylation of H3K28, which leads to a preferential differentiation of progenitors to the α‐ and β‐cell lineage at the cost of acinar cells. Pancreas‐specific conditional knockout of *Msk1* led to an increase in pancreatic α‐cell mass, and monoallelic loss of it increased β‐cell mass.^32^

Sirtuins are NAD(+)‐dependent deacetylases known to function in regulating cellular metabolism.^33^ Among them, Sirtuin 1 (Sirt1) has been shown to play an important role during the embryonic development in mice.^34^ Pancreas‐specific deletion of *Sirt1* impairs pancreatic β‐cell function, and its ectopic expression increases glucose tolerance. Moreover, it induces insulin secretion in response to glucose stimulation by posttranslational modification (deacetylation)of forkhead box protein A2 (FoxA2), a transcription factor regulating Pdx1, eventually leading to regulating Pdx1.^35^ FOXA2 facilitates the deposition of H3K4me1, a signature histone modification for open chromatin conformation, at the enhancer regions of some of the pancreas‐specific transcription factors (enhancer priming), including Pdx1 and thus regulates their expression.^36^ Another sirtuin, Sirt6, regulate pancreatic β‐cell function by regulating Thioredoxin interacting protein (Txnip), a thioredoxin‐binding protein which reduces the antioxidant activity of thioredoxin, involved in removing reactive oxygen species in cells. Its levels negatively correlate with GSIS in beta cells, and its overexpression could inhibit insulin secretion. The pancreatic β‐cells of *Sirt6*‐deficient mice showed decreased levels of glucose‐induced insulin secretion without any marked reduction in pancreatic β‐cell mass, probably suggesting a role for *Sirt6* in the regulation of glucose sensitivity of pancreatic β cells.^37^

The pancreas is a highly heterogeneous organ composed of different types of cells performing different functions. It is the center for the hormonal regulation of glucose homeostasis. Thus, a tight regulation of gene expression is required for the precise hormonal synthesis and secretion from the pancreas. Contrasting views are available regarding the epigenetic modifications of the genes encoding pancreatic hormones. An absence or low levels of active promoter modifications of the genes encoding islet‐specific hormones were reported, suggesting a role for distinct regulatory mechanisms.^38^, ^39^ Enhancer‐mediated regulation of islet‐specific hormones has also been shown using genome‐wide analysis of DNase I hypersensitive sites, analysis of histone H3 lysine methylation modifications (K4me1, K4me3, K79me2), and CCCTC factor (CTCF) binding sites in human islets. Identification of T2D‐associated single‐nucleotide polymorphisms (SNPs) mapping to these enhancers further fortifies their argument of enhancer‐mediated regulation of islet‐specific hormones.^38^ Bhandare et al.^39^ also observed significantly low levels of H3K4me2 and me3 at the promoters of *INS* and glucagon (*GCG*) in human islets. They found that in human pancreatic α‐cells, the promoter of MAF bZIP transcription factor (MAFB, an activator of glucagon gene) is occupied with H3K4me3 modification and leads to the activation of MAFB and eventually the glucagon gene, whereas in the β‐cells, the *PDX1* enhancer displayed enrichment of H3K4me1.^39^ Thus, the islet‐specific hormones are regulated by epigenetic modifications at the promoters of their transcription factors. Simultaneously, the promoter of yet another islet‐specific transcription factor, *NEUROG3*, showed enrichment of H3K27me3 concordant with its downregulation in adult pancreatic islets.^39^

**Figure 1:**
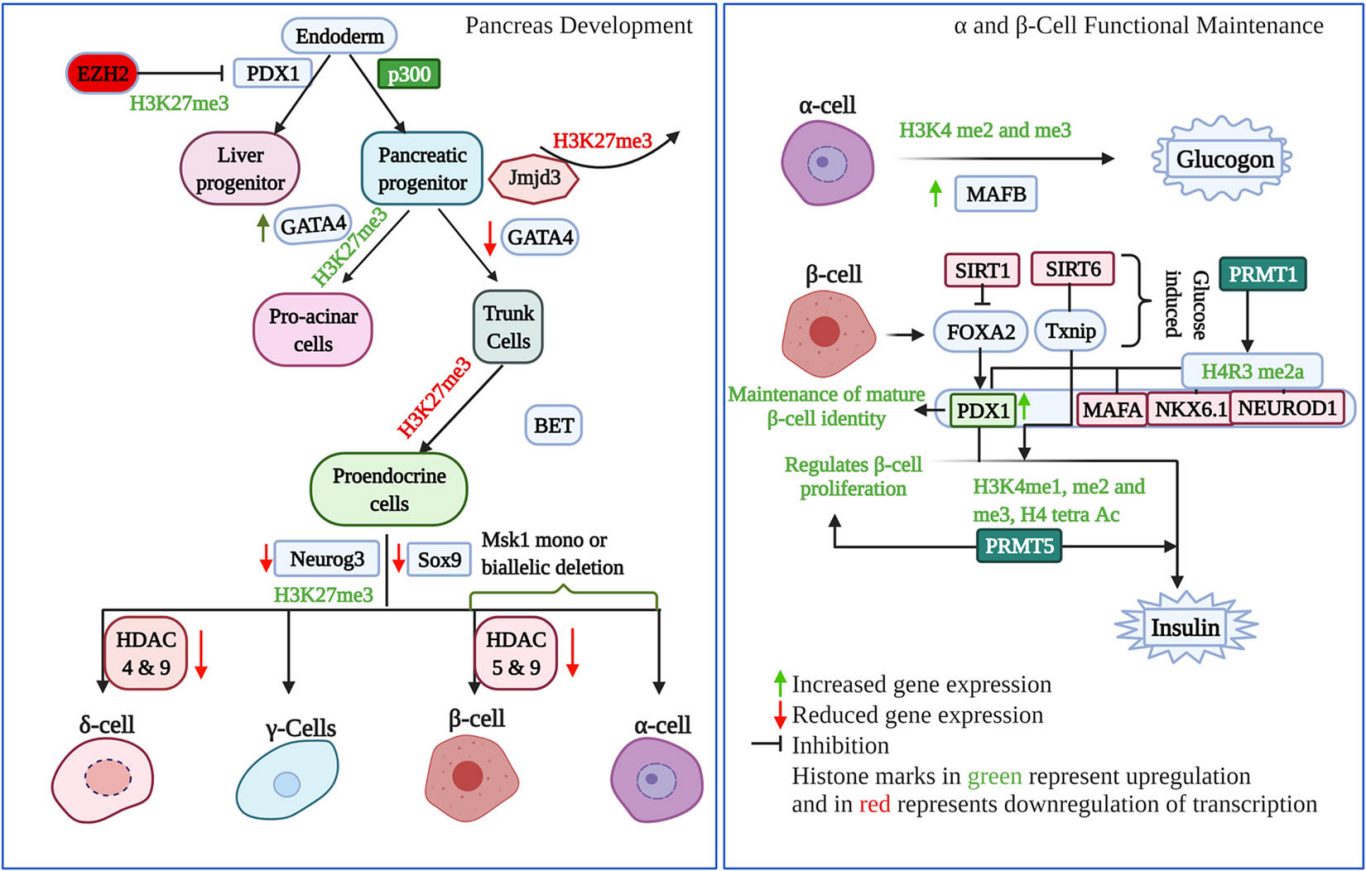
Schematic representation of the role of histone modifications in pancreas development and α and β cell function. On the left panel, the different histone modifiers and the known histone modifications with their effects on progenitor cell fates are indicated with arrows. The right panel shows the different histone modifications that regulate endocrine cell functions, mainly α‐ and β‐cells, which are the key regulators of insulin metabolism and signalling, Figure generated using BioRender. Red colour means the histone mark is removed, whereas green colour means the histone mark is added. Similarly, green arrows show upregulation, and red arrows show downregulation [Color figure can be viewed at wileyonlinelibrary.com]

However, several others have reported extensive histone modifications around pancreatic hormone loci and attributed these modifications to their precise regulation.^40^, ^41^ It has been reported that in islets, α‐cells show more bivalent (H3K4me3, transcriptional activation and H3K27me3, transcriptional repression) histone modifications in comparison to β‐cells and exocrine cells, whereas β‐cells displayed thousands of genes with monovalent (either H3K4me3 or H3K27me3) histone modifications, suggesting a role for epigenetic modification in the process of differentiation and dedifferentiation of pancreatic islet α‐ and β‐cells.^40^ Studies on the epigenetic modifications along the whole insulin locus in freshly isolated pancreatic β‐cells have suggested that H3K4me2 was uniformly enriched at the 11 kb region spanning *INS* gene compared to HeLa cells. Although H3K4me3 was also high in this region, it was more strongly associated with the INS promoter region. The distribution of H4 tetra‐acetylation (at lysines 5, 8, 12, and 16) and H3 di‐acetylation at this locus was similar to the H3K4me3 distribution. These open chromatin signatures extended from nearly 55 kb upstream to nearly 25 kb downstream of *INS*, suggesting that this entire region is in an open chromatin conformation in pancreatic β‐cells. Moreover, this region displayed low levels of H3K27me3, a signature of heterochromatin. The regulatory regions in the INS gene locus were associated with high levels of H3K4me1, a signature chromatin modification for enhancer regions.^41^ Another class of enzymes, protein arginine methyltransferases (PRMTs), alter gene expression through posttranslational modifications in histones and non‐histone proteins. Although nine PRMTs have been identified, PRMT1 catalyzes one‐third of the total arginine methylations in histone and nonhistone proteins.^42^ The genetic ablation of *Prmt1* in mice resulted in the loss of H4R3me2 and impairment of β‐cell function because of the restricted accessibility of key β‐cell transcription factors such as Nkx6.1, Mafa, Pdx1, and Neurod1.^43^ PRMT5 is known to control β‐cell proliferation in association with menin, a scaffold protein. The deletion of *Prmt5* in mice leads to reduced insulin expression, impaired glucose tolerance, and glucose‐stimulated insulin secretion (GSIS) through histone methylation‐related chromatin remodelling.^44^ Transcription factor, cAMP response element‐binding (CREB) regulates *Ins* and *Irs2* in β‐cells and stimulates β‐cell proliferation as well as insulin signalling.^45^ The role of histone modifications in pancreatic progenitor cell fate specification, differentiation and α and β‐cell function is summarized in Figure 1.

### 2.2 Histone modifications in T1D

Genetic and epigenetic factors are involved in the pathogenesis of T1D. Histone‐modifying enzymes play a critical role in regulating immune cell reactions, which contribute to the development of T1D. Comparative analysis of CD4^+^ T cells from peripheral blood of normal and T1D human samples suggested decreased HDAC1 expression in T1D samples and an impaired T cell function.^46^ Genes associated with autoimmune and inflammation‐related pathways, including CTLA4, transforming growth factor beta (TGFβ), nuclear factor kappa B (NF‐κB), p38 mitogen‐activated protein kinase, toll‐like receptor, and interleukin‐6 (IL‐6), showed increased H3K9me2 in lymphocytes from the peripheral blood of T1D patients compared to healthy controls, most likely because of the recruitment of EHMT2. This raised the possibility that these genes were actively transcribed at the onset of diabetes and were silenced by EHMT2/H3K9me2 recruitment because of feedback inhibition.^47^ Genome‐wide linkage analysis has identified genomic regions that make people susceptible to insulin‐dependent diabetes mellitus (IDDM) and has classified them into 18 IDDM groups. Human leukocyte antigen (HLA) genes such as *HLA‐DRB1* and *HLA‐DQB1* belonging to the IDDM1 group showed changes in H3K9ac in monocytes of T1D patients, resulting in altered expression of these proteins in them and thus altering their ability for antigen presentation.^48^ Cytotoxic T‐lymphocyte‐associated protein 4 (*CTLA4*) expression is upregulated in T1D because of changes in H3K9me2 at its T promoter region and dysregulation of CD8 T cell‐mediated immune reactions, leading to enhanced autoimmune reactions.^47^ Treatment with low doses of streptozotocin (STZ) causes damages to pancreatic β‐cells, resulting in the release of β‐cell‐specific antigens that eventually lead to autoimmune reactions and destruction of β‐cells by T cells infiltrating into pancreatic islets.^49^ HDACs are important for maintaining the tolerance against autoimmune destruction of β‐cells. Experiments have shown that mice with pancreatic β‐cells lacking Hdac3 displayed decreased pancreatic insulin content and disrupted glucose‐stimulated insulin secretion, with intermittent spontaneous diabetes and enhanced susceptibility to STZ‐induced diabetes.^50^ However, treatment with BRD3308, an Hdac3 inhibitor, protected rats against T1D by reducing immune cell infiltration into pancreatic islets suggesting a role for HDACs in the establishment of self‐tolerance.^51^ Treatment with HDAC inhibitors such as sodium butyrate (NaB) and N‐(2‐amino‐phenyl)‐4‐(4‐pyridin‐3‐yl‐pyrimidin‐2‐ylamino)‐methyl)‐benzamide (MGCD0103) has been found effective in improving blood glucose and insulin levels in STZ‐induced diabetic rat models.^52^, ^53^, ^54^

### 2.3 Histone modifications in T2D and GDM

T2D is characterized by a decrease in β‐cell mass, impaired insulin secretion, and insulin resistance resulting from impaired β‐cell function. Although more than 70 loci have been shown to be associated with increased T2D risk by genome‐wide association studies, this explains only ~11% of all T2D cases.^55^ This indicates the significance of epigenetic factors and the resulting alterations in gene expression in the incidence of T2D.

HDACs regulate pancreatic endocrine function, and glucose homeostasis and their dysregulations have been shown to be associated with T2D. For example, increased expression of *HDAC7* was observed in pancreatic β cells from T2D patients. Insulin signalling controls pancreatic β cell mass and function by increasing β cell division and insulin secretion.^56^, ^57^ HDACs control IRS2 dependent insulin signalling through the depletion of H3K9 acetylation of *IRS2* promoter resulting in repression of *IRS2* expression. Treatment with HDAC inhibitors increased lysine acetylation of *IRS2* with a concomitant decrease in phosphorylation of tyrosines of IRS2 protein.^58^ Therefore, HDAC inhibitors have an enormous potential to emerge as an option for treating T2D. For example, treatment with 3‐[5‐(3‐(3‐Fluorophenyl)‐3‐oxopropen‐1‐yl)‐1‐methyl‐1H‐pyrrol‐2‐yl]cf‐N‐hydroxy‐2‐propenamide (MC1568), a selective Class II (IIa) histone deacetylase (HDAC II) inhibitor, improved impairment of insulin secretion associated with T2D in the islets of T2D patients.^59^ A study using the intrauterine growth retardation model showed that at the embryonic stage, *Pdx1* gene expression was downregulated in growth‐retarded rats because of the loss of Usf1 (transcription factor regulating *Pdx1*) binding at the *Pdx1* proximal promoter region, Hdac1 recruitment to the promoter, and deacetylation of H3 and H4 at the *Pdx1* promoter in the pancreatic β‐cells. Postnatally, the *Pdx1* promoter region undergoes H3K4 demethylation and H3K9 methylation, and in adulthood, CpGs at the *Pdx1* proximal promoter region is methylated, leading to the permanent silencing of *Pdx1*.^60^ FoxO1 is a key regulator of Pdx1, which is needed for β‐cell development and glucose homeostasis. Acetylated FoxO1 represses its target genes by binding to their promoters.^61^ Deacetylation of FoxO1 by Sirt6 increases the expression of *Pdx1* and *Glut2*.^62^ Sirtuins (SIRTs) are Sir2 protein homologs that function as H3K9 deacetylases. In addition to their roles in various processes such as stress resistance, apoptosis and regulating life span, they also play a significant role in regulating metabolism. Sirt6 controls the expression of the critical transcription factor, Hypoxia‐inducible factor 1‐alpha (Hif1α) that regulates the selection of oxidative phosphorylation versus glycolytic pathway under conditions of nutrient stress. Under normal glucose conditions, at the promoter region of the key genes regulating glycolysis, Sirt6 may be competing out Hif1α and keeps those genes in a repressed state and maintains the influx of glucose towards mitochondrial oxidative respiration. It is also possible that Sirt6 regulates the activity of Hif1α and thereby represses the Hif1α target genes. Thus Sirt6 acts as one of the master regulators of glucose homeostasis.^63^ Acetylated H2A.Z and H3K9/14 acetylation at the promoter of hexokinase 2 (*HK2*) increased its expression by twofolds in response to insulin. Similarly, treating rat L6 myotubes with insulin repressed IRS2 and PIK3R2 expression and treatment with trichostatin, a potent and reversible inhibitor of histone deacetylase, resulted in increased histone acetylation at the promoters of *Irs2* and *Pik3R2* and increased their expression.^64^ HDAC6 impairs glucocorticoid‐induced hepatic glucose homeostasis by overexpressing *Pepck, G6P* and pyruvate carboxylase by changing H4K8 acetylation at the PEPCK gene promoter. HDAC6 regulates glucocorticoid‐induced hepatic gluconeogenesis by controlling the acetylation of Hsp90 and the subsequent nuclear translocation of the glucocorticoid receptor (GR). Deacetylated Hsp90 binds to GR and for a nuclear translocating complex. Deletion of HDAC6 or inhibition by tubacin, a hydroxamic acid HDAC inhibitor that inhibits only HDAC6 activity, ameliorates glucose homeostasis.^65^ T2D patients showed elevated levels of SIRT5 in serum. Further investigations using pancreatic cell lines, MIN6 (mouse) and INS‐1 (rat), suggested that SIRT5 might regulate the PDX1 gene by deacetylation of H4K16 at *PDX1* promoter. Inhibiting the deacetylation in MIN6 and INS‐1 cell lines by valproic acid (VPA), an HDAC inhibitor, increased *PDX1* expression and insulin secretion.^66^

HMT also play a role in the pathogenesis of T2D. Mll2 is an H3K4 methyltransferase that participates in the mono‐, di‐, and trimethylation of H3K4. Haploinsufficiency of Mll2 led to hyperglycemia and hyperinsulinemia at fasting and impaired glucose tolerance, and blunted insulin secretion in response to glucose loading. Haploinsufficient mice for Mll2 showed peripheral insulin resistance. Genes involved in β‐cell regulation, glucose metabolism, and fatty acid transport, such as *Neurod1*, neuronatin (*Nnat*), ectonucleotide pyrophosphatase/phosphodiesterase 1 (*Enpp1*), *Enpp2*, phosphatidylinositol specific phospholipase c x domain containing 1 (*Pcldx1*), and solute carrier family 27 member 2 (*Slc27a2*) were downregulated in these mice.^67^ In humans, MLL2 has been shown to interact with menin (MEN‐1), mutations in which are responsible for multiple endocrine neoplasias, to regulate the pancreatic β cell proliferation by downregulating the expression of the cell cycle inhibitors such as CDKN1A, CDKN1B, and CDKN2C.^68^ However, further studies are required to understand the role of MLL2 in human diabetes.

H3K9 methylations mark heterochromatin formation and transcriptional gene silencing and thus play a major role in controlling gene expression. Histone methyltransferase and demethylase enzymes regulate the dynamics of histone methylation. JmjCdomain‐containing histone demethylase 2A (Jhdm2a) catalyzes the removal of H3K9 methylation marks and regulates peroxisome proliferator‐activated receptor α (Pparα) and Apolipoprotein C1 (ApoC1), which regulate energy homeostasis, including adipogenesis, regulation of fat storage and glucose transport. The absence of Jhdm2a leads to obesity, hypertriglyceridemia, hypercholesterolemia, hyperinsulinemia, and hyperleptinemia in mice.^69^ H3K36me2, a transcriptional activation mark at the promoter regions of *Pdx1*, is regulated by the histone methyltransferase, Nuclear receptor‐binding set domain protein 2 (NSD2), and the downregulation of NSD2 expression can lead to T2D.^70^ Treatment with Metformin, a first‐line medication used for the treatment of T2D, reverses H3K36me2 marks in T2D patients.^71^ Similarly, the expression of *Glut4*, the cellular glucose transporter, is regulated by trithorax domain‐containing lysine methyltransferase 7 (SET7).^72^

Exercise is known to protect against T2D by inducing epigenetic mechanisms to upregulate the expression of the glucose transporter *Glut4*. Glut4 activates calmodulin kinase II (CaMKII), leading to histone acetylation at the NRF2‐binding regions of the promoter of *Mef2*, the major transcription regulator of *Glut4*, leading to the upregulation of *Mef2* and eventually *Glut4*.^73^ Diet‐induced obesity (DIO) is often correlated with the incidence of T2D in rodent models, and epigenetic changes have been proven as one of the major reasons for this. It has been shown that DIO leads to an increase in H3K27 acetylation at 13,369 cis‐regulatory elements and a decrease in 4610 cis‐regulatory elements in pancreatic β cells in mice. Increased acetylation was found at*Nrf1*, GA binding protein transcription factor subunit alpha (*Gabp*α), and Mef2A‐binding regions of DNA, and decreased acetylation was found at the MAFK binding region, a known negative regulator of β‐cell function.^74^ Obesity induces chronic inflammation and subsequent activation of the immune system, causing the development of insulin resistance and T2D. Coactivator‐associated arginine methyltransferase 1 (CARM1)/protein arginine methyltransferase‐4 (PRMT4), an epigenetic modifier enzyme causing protein methylation, the expression has been found to regulate the expression of proinflammatory cytokines. Its expression is positively correlated with the expression of the gastric inhibitory polypeptide, IL‐4, IL‐7, IL‐13, IL‐17, fibroblast growth factor (FGF basic), granulocyte colony‐stimulating factor, interferon‐gamma, and tumour necrosis factor alpha (TNFα) in T2D patients. Thus, the increased expression of CARM1 may account for the increased expression of proinflammatory markers in obesity‐induced T2D.^75^

**Figure 2:**
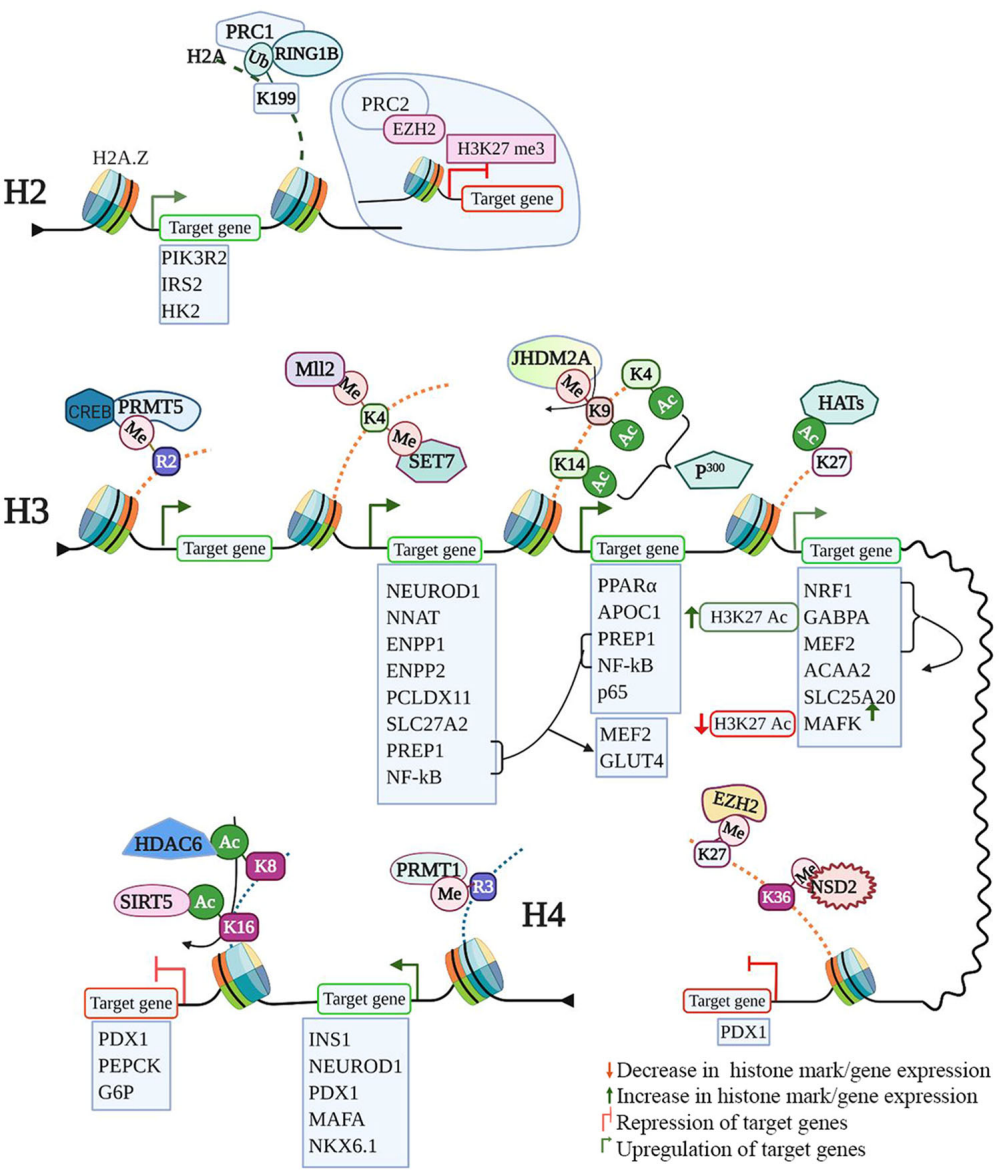
Histone modifications in various aspects of pancreatic development and pathogenesis of diabetes. H2A.Z serves as a histone mark, which upregulates gene expression by facilitating chromatin access to the transcription machinery. The monoubiquitination of K199 by PRC1 facilitates chromatin compaction by further recruiting PRC2, which, in turn, catalyzes H3K27Me3, a gene repression mark. H3K27Me, H3K9Me, and H3K36Me serve as histone marks of target gene repression at gene promoters, whereas H3K4Me, H3R2Me, and H3Ac promote target gene activation. All these marks are altered in diabetes. H4R3 methylation by PRMT1 activates target gene expression. H4K8 and H4K16 acetylation's target genes expressions are targets of Class II and Class III HDACs. Target gene boxes with green outlines show gene activation, and the ones with red outlines show gene repression. Figure generated using BioRender [Color figure can be viewed at wileyonlinelibrary.com]

Although only a few studies have been conducted regarding histone modifications associated with GDM, it is known that epigenetic changes induced during pregnancy can induce GDM and lead to congenital diseases in offspring. A comparison of placentas from GDM and control mothers showed that GDM babies had reduced H3K9ac expression, although H3K4me3 expression was not significantly different.^76^ Some of the modifications and the targets changed by histone tail modifications in diabetes are shown in Figure 2, and the list of genes is given in Table 1.

**Table 1:** List of genes those are linked to dabetes and the associted histone modifications

Tissue, gene/locus	Modification	Effect on gene expression	References
Mice, global	Global histone demethylation due to MLL2 haploinsufficiency	Hyperglycemia, hyperinsulinemia, impaired glucose tolerance and peripheral insulin resistance. Downregulation of genes involved in β‐cell regulation, glucose metabolism, and fatty acid transport such as *Neurod1*, *Nnat*, *Enpp1*, *Enpp2*, *Pcldx1*, and *Slc27a2*	^67^
Mice, global	Increase in H3K9Me1 and H3K9Me2 due to the absence of Jhdm2a demethylase	Downregulation of PPARα and ApoC1, Obesity, hypertriglyceridemia, hypercholesterolemia, hyperinsulinemia, hyperleptinemia	^69^
Mice, global	Increase in H3K27Ac at cis‐regulatory elements	Upregulation of global gene expression	^74^
Human, global	Upregulation of CARM1	Upregulation of FGF, G‐CSF, IFNγ, and TNFα	^75^
Human, pancreatic β cells	HDAC7 upregulation	Impaired glucose‐induced insulin secretion	^58^
Mouse MIN6 cells, *Irs2*	Hdac6 mediated H3K9 deacetylation	Downregulation of *Irs2*	^65^
Mouse pancreatic β cells	Sirt6 mediated deacetylation of FoxO1	Downregulation of *Pdx1* and *Glut1*	^62^
IUGR rat models/pancreatic β cells	Hdac1 mediated H3 and H4 deacetylation	Downregulation of *Pdx1*	^60^
Mouse liver/nuclear translocation of GR	HDAC6 mediated deacetylation of HSP90 due to increased insulin	Increased gluconeogenesis	^65^
Mouse, skeletal muscles	SET7 methyltransferase and p300 acetyltransferase mediated modifications	Regulation of Prep1, a transcription factor for *Glut4*	^72^
Rat muscle satellite cell line L6	H2A.Z accumulation and increased acetylation of H3K9/14 due to increased insulin	Upregulation of *Hexokinase 2*	^64^
Rat, muscles	Exercise‐induced CaMKII mediated histone acetylation	Increased expression of the TF *Mef2* and its target *Glut4*	^73^

## 3 DNA METHYLATION

The failure to maintain true epigenetic marks leads to changes in the genetic information of the cell and inappropriate gene expression, resulting in different diseases.^77^ In normal mammalian cells, over 80% of CpG dinucleotides are methylated, which, in turn, are suggested to play critical roles in maintaining the integrity of the chromatin structure and the regulation of transcription.^78^ Chromatin replication during the synthesis (S) phase of the cell cycle leaves the newly synthesized DNA strand without cytosine methylation marks found in parental cells, which needed to be maintained. This cytosine methylation of DNA is faithfully preserved in mammalian cells between successive cell divisions by DNA (cytosine‐5) methyltransferases (DNMTs). Three DNMTs, namely, DNA methyltransferase 1 (DNMT1), DNA methyltransferase 3a (DNMT3a), and DNA methyltransferase 3b (DNMT3b), predominantly perform these functions in eukaryotic cells. Of these, DNMT1 has a strong preference for hemimethylated DNA in vitro.^79^ Previous studies have shown that the DNMT1/UHRF1/PCNA complex is stably associated with the newly replicated origin in the mammalian cells and methylates the newly synthesized daughter strands after replication by reading the methylation pattern of the parental strand.^80^ DNMT3a and DNMT3b are known to act de novo, especially during early embryonic development, and establish novel methylation patterns in the genome.^81^ DNA methylation, catalyzed by DNMT1, DNMT3a, and DNMT3b, involves donating a methyl group from S‐adenosyl methionine to the 5′‐position of a cytosine nucleotide linked to a guanine nucleotide (CpG dinucleotide) by a phosphodiester bond. Regions with a high content of CpG dinucleotides, called CpG islands, are found in the regulatory regions of many genes, including promoters and enhancers. Methylation at CpG islands is highly dynamic in nature. An increase in methylation is referred to as hypermethylation, and it usually leads to the downregulation of gene expression. A decrease in methylation is referred to as hypomethylation, which leads to the upregulation of gene expression.^82^, ^83^ An enzymatic pathway mediates the demethylation of methylated DNA and members of ten‐eleven translocation (Tet family) enzymes catalyze this activity. They mediate the conversion of methyl cytosines to hydroxymethyl cytosines, which are eventually replaced by unmodified cytosine by a base excision repair mechanism.^84^

There are several ways by which DNA methylation regulates gene expression. Interaction between Dnmts and histone‐modifying enzymes such as HMTs and HDACs could suppress gene repression. Dnmt1 and Dnmt3a bind to SUV39H1, a HMT that methylates H3K9 and consequently restricts gene expression.^85^ The transcriptional gene silencing effect of DNA methylation is partly mediated through the recruitment of HDAC inhibitors also.^86^

### 3.1 DNA methylation in the development of the pancreas

Development‐related differentially methylated regions (dDMRs) undergo dynamic changes during the development of the pancreas. In humans, at 22 weeks of development (corresponding to the second trimester of development, when pancreatic cell lineages have wholly developed and tip‐trunk compartmentalization has been completed^87^), an increase in methylation was observed at 449 dDMRs, and loss of methylation was observed in 61 dDMRs. dDMRs at 6‐phosphofructo‐2‐kinase/fructose‐2,6‐biphosphatase 3 (*PFKFB3)*, involved in insulin secretion and at the proximal promoters of aminoacylase 3 (*ACY3*), hepatocyte nuclear factor 1‐alpha (*HNF1A*), and hepatocyte nuclear factor 4‐alpha (*HNF4A*), genes with key roles in pancreas development, were among the hypomethylated group, whereas the promoter region of NK6 homeobox 1 (*NKX6*.1), an important gene in β‐cell development, was among the hypermethylated dDMR group.^88^ The absence of *Dnmt1* in the pancreatic progenitors during the early stages of development greatly inhibited the development of the pancreas. In *Dnmt1* knockout mice, pancreatic progenitor cells were arrested at the G2/M stage of the cell cycle and were removed by apoptosis,^89^ which was also observed in zebrafish.^90^ CpG methylation at the promoter region of the aristaless‐related homeobox (Arx) gene was shown to regulate the cell fate determination of pancreatic progenitor cells and the identity of the differentiated progenitor cells. Hypermethylation of the*Arx* promoter region favoured β‐cell differentiation, whereas hypomethylation favoured α‐cell differentiation.^91^

Conditional deletion of *Dnmt1* expression in immature β‐cells results in ectopic expression of *Arx* and cell fate conversion of β‐cells into α‐cells, resulting in glucose intolerance.^92^ Homeobox containing transcription factor Nkx2.2 recruits a repressive complex that contains Hdac1 and Dnmt3a specifically to the*Arx* promoter in β‐cells to suppress *Arx* expression to maintain β‐cell identity. Disruption of this complex results in ectopic *Arx* expression in β‐cells, leading to β‐ to α‐cell conversion and eventually leading to diabetes.^93^ An investigation for CpG methylations at the *INS* locus in freshly isolated human pancreatic β‐cells identified two positions (2190533 and 2133296) that are free of methylation and are enriched with active histone modifications, suggesting that these could be regulatory elements for the gene cluster at the *INS* locus or a boundary element for open chromatin at the *INS* locus.^41^ Together, these studies demonstrated that the epigenetic marks of CpG methylation are critical for early pancreatic development and their function and play an important role in maintaining the versatility of cell types in pancreatic islets.

### 3.2 DNA methylation in Type 1 diabetes

Over the last decade, there was a drastic increase in the incidence of T1D, and even monozygotic twins only show 40% concordance for T1D occurrence, suggesting a role for environmental factors and epigenetic mechanisms in the aetiology of the disease.^94^ Immunological destruction of pancreatic β‐cells leads to T1D. This is initiated by the infiltration of CD4 and CD8 T cells into the pancreas in response to the cytokines secreted by T cells and antigen‐presenting cells because of exposure to autoantigens. Epigenetic mechanisms could account for this breakage of self‐tolerance. One of the first genes that were studied for promoter methylation was *INS*, and of the seven CpG sites close to its transcription start sites verified, it was found that in the pancreatic islets of T1D donors, hypomethylation occurred at CpGs at −19, −135, and −234, whereas hypermethylation occurred at −180. No changes in the methylation pattern were observed at −69, −180, and −206 compared to the controls. In these studies, the extent of CpG methylations was not correlated to the levels of glycated haemoglobin or the duration of T1D.^95^ Comparative analysis of methylation at differentially variable CpG positions between identical twins discordant for incidence of T1D, followed by the analysis of gene regulatory networks, has identified enrichment of pathways involved in immune cell metabolism and the cell cycle, including mammalian target of rapamycin signalling.^96^ A similar study using peripheral lymphocytes isolated from three pairs of monozygotic twins discordant for T1D and six pairs of monozygotic twins concordant for T1D showed altered methylation at 88 CpG sites in, genes including *HLA*, *INS*, *IL2RB*, and *CD226*, most of which are critical for initiating immune reactions against autoantigens.^94^ T1D‐multiple variable positions (MVPs) arise very early in the etiological process, which leads to overt T1D.^97^ A recent study on global DNA methylation in one set of monozygotic quadruplets discordant for T1D showed MVPs in diabetes‐associated genes (*INS*‐*IGF2*, *SH2B3*, *MEG3*, and *ORMDL3*) in the affected twins.^98^ A study using lymphocytes infiltrating pancreatic islets in NOD mice indicated that a cytokine‐induced change in methylation marks in the exon 1 region of both *Ins1* and *Ins2* genes changed over time during the development of T1D.^99^ Alterations in the methylation status of CpG islands in integrin subunit beta 3 binding protein (*ITGB3BP*), AF4/FMR2 family member 3 (*AFF3*), protein tyrosine phosphatase non‐receptor type 2 (*PTPN2*), cathepsin H (*CTSH*), and cytotoxic T‐lymphocyte associated protein 4 (*CTLA4*) genes in human peripheral blood lymphocytes also increased the risk of T1D.^100^ Major histocompatibility class (MHC) proteins are involved in antigen presentation, thereby inducing immune reactions against both alloantigens and autoantigens.^101^ Changes in the methylation of genes encoding MHC proteins and subsequently reduced expression of human leukocyte antigen (HLA‐DR) protein and extensive cis‐metQTLs (methylated quantitative trait loci, genetic loci associated with site‐specific DNA methylation of CpGs) were also identified in T1D patients.^102^

### 3.3 DNA methylation in Type 2 diabetes

T2D is a complex metabolic disease involving dysregulated expression of multiple genes involved in oxidative phosphorylation; carbohydrate, amino acid, lipid metabolism; inflammation; and glycan degradation.^103^ Genome‐wide genetic variations, epigenetic variations, and environment interactions influence gene expression and islet function and increase the risk of developing diabetes.^103^, ^104^ The downregulation of metabolic genes often characterizes T2D. Downregulation of PGC‐1α was observed in the skeletal muscles and pancreatic islets of T2D patients. Barres and colleagues attributed this to non‐CpG methylation at the promoter region of peroxisome proliferator‐activated receptor‐gamma co‐activator 1‐alpha *(PPARGC1A)* caused by DNMT3B. Non‐CpG methylations are a significant but smaller fraction of nucleotide methylations seen in the CpT, CpA, and CpC asymmetric (non‐CpG) dinucleotides. It has been shown that these non‐CpG methylations caused a downregulation in the expression of PGC‐1α in skeletal muscle cells and myotubes upon exposure to TNFα or free fatty acids but not insulin or glucose.^105^ Similarly, in pancreatic islets of T2D donors, the downregulation of PGC‐1α was partially caused by CpG methylation at the promoter region of *PPARGC1A*.^106^ T2D donors display reduced insulin secretion because of the downregulation of *INS* expression. The reduced insulin expression in pancreatic islet cells of T2D donors was shown to correlate with the methylation at four CpG sites located 234, 180, and 102 bp upstream and 63 bp downstream of the transcription start site (CpG −234, −180, −102, and +63, respectively) of the insulin gene.^107^ When human pancreatic islets exposed to hyperglycemic conditions in vitro were examined, altered methylation was observed at CpG sites of genes involved in the TGFβ signalling pathway, Notch signalling pathway, and SNARE interactions in vesicular transport, such as glycine receptor alpha 1 (*GLRA1*), dexamethasone‐induced ras‐related protein 1 (*RASD1*), *VAC14*, solute carrier organic anion transporter family member 5A1 (*SLCO5A1*), and cholinergic receptor nicotinic alpha 5 subunits (*CHRNA5*). An increase in methylation at the CpG sites of the *PDX1* gene was also observed when these islets were exposed to high amounts of glucose.^108^ Large‐scale genome analysis for methylation has led to the identification of differential methylation in the pancreatic islets between T2D patients and controls. One such study identified 1649 CpG sites annotated to 843 genes. Three genes, *CDKN1A*, *PDE7B*, and *SEPT9*, displayed upregulation of their expression in islets from T2D patients concordant with the hypomethylation of CpGs at their promoters. Overexpression of some of these genes led to impaired glucose‐stimulated insulin secretion or β‐cell proliferation, confirming the role of their dysregulated expression in the pathogenesis of T2D.^109^ Alterations in CpG methylation levels in the regulatory regions of many of the genes related to metabolic pathways such as thioredoxin interacting protein (*TXNIP*),^110^ solute carrier organic anion transporter family member (*SLC30A8*),^111^ GLP1 receptor (*GLP1R*),^112^ peroxisome proliferator‐activated receptor gamma (*PPARG*), potassium voltage‐gated channel subfamily q member 1 (*KCNQ1*), transcription factor 7‐like 2 (*TCF7L2*), and insulin receptor 1 (*IRS1*) have been observed in T2D patients.^113^

Whole‐genome bisulfite sequencing of human pancreatic islet cells identified 457 genes, including nuclear receptor subfamily 4 group A member 3 (*NR4A3*), Parkinson disease‐2 (*PARK2*), phosphotyrosine interaction domain containing 1 (*PID1*), solute carrier family 2 member 2 (*SLC2A2*), and suppressor of cytokine signalling 2, that had both DMRs and associated changes in gene expression in T2D.^114^ These DMRs contain binding sites for islet‐specific transcription factors such as PDX1, TCF7L2, adenylate cyclase 5 and enhancer regions driving islet‐specific gene expression. DNA methylation at the *KCNQ1* locus, a region in the human genome considered to be highly associated with the risk of T2D development, was inversely associated with insulin sensitivity and serum adiponectin. A SNP at rs231840 in this region ablated this association, suggesting that this site regulates insulin sensitivity and adiponectin levels through CpG methylation.^115^ A genome‐wide association study of more than 15,000 nondiabetic Europeans clarified the role of differential methylation and gene expression in the early pathogenesis of T2D. It identified that fasting insulin levels are correlated to methylation levels at CpGs in leucine zipper and EF‐hand containing transmembrane protein 1 (*LETM1*), RNA‐binding motif protein 20 (*RBM20*), *IRS2*, mannosidase alpha Class 2a member 2 (*MAN2A2*), and the 1q25.3 regions of the genome. It also identified that methylations at CpGs in Fc receptor‐like 6 (*FCRL6*, increased with fasting blood glucose), signalling lymphocytic activation molecule family member 1 (*SLAMF1*), apolipoprotein B mRNA‐editing enzyme catalytic polypeptide 3 (*APOBEC3H*), and the 15q26.1 region are associated with blood glucose levels. Further, in silico analysis predicted a connection between the expression of these genes and the adaptive immune system.^116^ A high‐fat diet (HFD) often induces the development of T2D by inducing insulin resistance. A recent study showed that feeding mice with a HFD‐induced altered methylation at the enhancer and promoter regions of the Ankyrin repeat domain 26 (*Ankrd26*) gene, a gene associated with the development of T2D, leading to its downregulation.^117^ Ankrd26 controls organ size, and its absence has been shown to cause hyperphagia, downregulation of *Pparγ*, and age‐dependent fat accumulation in mice.^118^ Apart from genes, methylation alterations at the noncoding genomic elements may also lead to T2D by affecting their gene regulatory functions as enhancers or boundary elements. A 14‐year‐long cohort study with T2D individuals revealed that global LINE‐1 DNA methylations (LINEs are long interspersed nuclear elements that show transposon activity) affect specific metabolic markers.^119^ The enhancer functions of LINEs are possibly affected by methylation, which eventually leads to the downregulation of the genes under their regulation.

T2D, a complex metabolic disease, is known to affect metabolic tissues other than the pancreas, such as the liver, muscles, and adipose tissues.^103^, ^120^ T2D patients often develop hepatic insulin resistance, eventually developing the nonalcoholic fatty liver disease. Studies have revealed that the hypomethylation of the platelet‐derived growth factor‐alpha gene (*PDGFA*) and the consequent enhancement in its expression observed in T2D patients have been associated with hyperinsulinemia, increased insulin resistance, and increased steatohepatitis risk.^120^ Similarly, another study found hypomethylation of ATF‐binding motifs of genes involved in insulin resistance and hepatic glycolysis, with a concomitant increase in their mRNA expression in T2D‐affected men.^121^ Nilsson and colleagues studied insulin resistance in hepatocytes of T2D patients using the liver biopsies of 35 T2D samples and 50 control samples and found that T2D leads to the hypomethylation of 94% of the differentially methylated CpG sites and only 6% showed hypermethylation. They attributed the reduction in methylation to the reduced erythrocyte folate levels in the T2D samples.^122^ Hepatic glucose production (HGP) is the physiological process by which the liver controls the glucose levels in the blood by maintaining a balance between gluconeogenesis and glucagon‐induced glycogenolysis.^123^ Dysregulated HGP due to insulin–glucagon imbalance is often associated with T2D. This is caused by low insulin levels as well as increased insulin resistance in hepatocytes in T2D patients. Tet3‐mediated demethylation of the promoter region of the *HNF4*A gene and the consequent upregulation of its fetal version, the P2 isoform, have been attributed to the dysregulation of HGP in T2D patients.^124^

A study conducted among individuals with a family history of T2D revealed altered DNA methylation of genes in biological pathways, including mitogen‐activated protein kinase (MAPK), insulin, and calcium signalling in their muscle tissues. Moreover, individual genes such as *MAPK1*, *MYO18B*, *HOXC6*, and the AMP‐activated protein kinase subunit *PRKAB1* with known function in muscles, such as the formation of sarcomeres, displayed differential methylation in men with the family history of T2D compared to men without any family history of T2D.^125^ Another study conducted in male twins aged 53–80 years, discordant for T2D, showed that acquired DNA methylation changes in skeletal muscle or adipose tissue gene promoters are quantitatively small between Type 2 diabetic and nondiabetic twins. The major genes that showed hypermethylation were *PPARGC1A* in muscles and *HNF4A* in adipose tissue.^126^ *HNF4A* has been shown as a regulator of fatty acid metabolism in hepatocytes through the regulation of PPARγ and PPARα expression.^127^ Hypermethylation of *PPARGC1A* was also reported in the subcutaneous adipose tissue of low birth weight (LBW) men who tended to develop T2D.^128^ In another study, when the DNA methylation profile dataset GSE38291, downloaded from the Gene Expression Omnibus database, was examined for changes in DNA methylation of gene promoters associated with T2D by GO analysis, a total of 38 genes (e.g., *SIRT1*, N‐acetyltransferase 6 [*NAT6*], phospholipase A2 group XIIB [*PLA2G12B*], and nuclear factor of activated T cells calcineurin‐dependent 1 [*NFATC1*]) were identified to be differentially methylated between the muscles of T2D and control samples. Analysis of transcription factor binding sites for the methylation changes revealed that the binding sites of zinc finger E‐box binding homeobox 1 (*ZEB1*) were hypermethylated, whereas the binding sites of three TFs (methyl CpG binding protein 2 [MECP2], TFEB, and TFAP4) were significantly hypomethylated.^129^

### 3.4 DNA methylation in gestational diabetes

GDM, which has a high incidence and adversely affects the quality of life of pregnant women during pregnancy, is also known to affect fetal health. Studies show that epigenetic changes play a major role in the development of GDM. The methylation status of the placenta plays a key role in maintaining glucose homeostasis phenotype among mothers. It regulates the “maternal‐fetal conflict” for nutrient shunting by choosing between the methylation of maternal and paternal alleles. Maternal and paternal alleles in the placenta are imprinted for resolving this conflict in an amicable way for both the mother and the offspring. In the placenta, the genes that are expressed from paternal alleles act to allocate more nutrients to the maintenance of the fetus, while the gene expression coordinated by maternal alleles helps the mother to conserve her resources.^130^ The effects of DNA methylation on GDM are usually studied using the Infinium 450k bead array or MeDIP of the placenta. Genes involved in the glucose metabolism of energy metabolism pathways showed altered methylation following GDM incidence.^131^, ^132^, ^133^, ^134^, ^135^ Some of the genes that showed altered methylation were retinol‐binding protein 4 (*RBP4*), *GLUT3*, *Resistin*, *PPAR*α,^132^ purinergic receptor 5 (*P2RX5*), *CCDC15*, and *ADAM12*.^133^ Alexander and colleagues^131^ reported gender‐specific changes in methylation patterns in the offspring of GDM mothers. They showed that female offspring display an increase in methylation compared to males, resulting in the downregulation of piwi‐like RNA‐mediated gene silencing 3 (*PIWIL3*), cytochrome b‐245 alpha chain (*CYB*α), glutathione s‐transferase mu 1 (*GSTM1*), *GSTM6*, potassium voltage‐gated channel subfamily E regulatory subunit 1 (*KCNE1*), and nucleoredoxin (*NXN*) expression. A study to underpin the effects of GDM on birth weight of offspring, and maternal GDM on the development of T2D, identified 75 differentially methylated CpG loci compared to normal offspring, representing 72 genes with relevance to altered growth and metabolism.^134^ Hypomethylation of CpG islands at promoter regions of cyclin‐dependent kinase inhibitor 2A (CDKN2A/B), and proapoptotic genes, have been observed in the offspring of mothers with GDM leading to apoptosis in pancreatic islet cells and eventually leading to T2D in adulthood.^135^ Methylation at the promoter region of *PPARGC1A* and the corresponding reduction in its expression was linked to the abnormal glucose metabolism in the offspring of GDM mothers.^37^ In mice, the offspring of GDM mothers showed alterations in the methylation pattern of the DMRs of the ankyrin repeat and ph domain 2 (*Agap2*), 1‐phosphatidylinositol‐4,5‐bisphosphate phosphodiesterase beta‐1 (*Plcbr1*), *Hnf1b*, guanine nucleotide‐binding protein, alpha stimulating (*Gnas*) fructose‐bisphosphatase 2 (*Fbp2*), cadherin‐13 (*Cdh13*), wingless‐type 2 (*Wnt2*), *Kcnq1*, luteinizing hormone/choriogonadotropin receptor (*Lhcgr*), and iroquois‐class homeodomain protein (*Irx3*) genes, which eventually led to increased susceptibility to T2D in adulthood.^136^ The disposition of the offspring to T2D risk due to unhealthy maternal diet habits during pregnancy has also been known to be mediated through alterations in the CpG methylation patterns of insulin expression or insulin‐signalling related genes.^137^ A study conducted on pregnant women who were overweight or obese indicated that the methylation level of cg05009389 in the 3′‐untranslated region (UTR) of perilipin 1 (*PLIN1*) was negatively correlated with maternal weight and an increase in insulin levels during gestation. The methylation levels of cg17586860 and cg18197392 in the 5′‐UTR region of somatostatin receptor 4 (*SSTR4*), an insulin resistance‐related gene, were negatively correlated with changes in dietary carbohydrate intake and glycemic load across gestation, whereas cg14631053 methylation positively correlated with mRNA expression of *SSTR4* in the placenta.^138^

**Figure 3:**
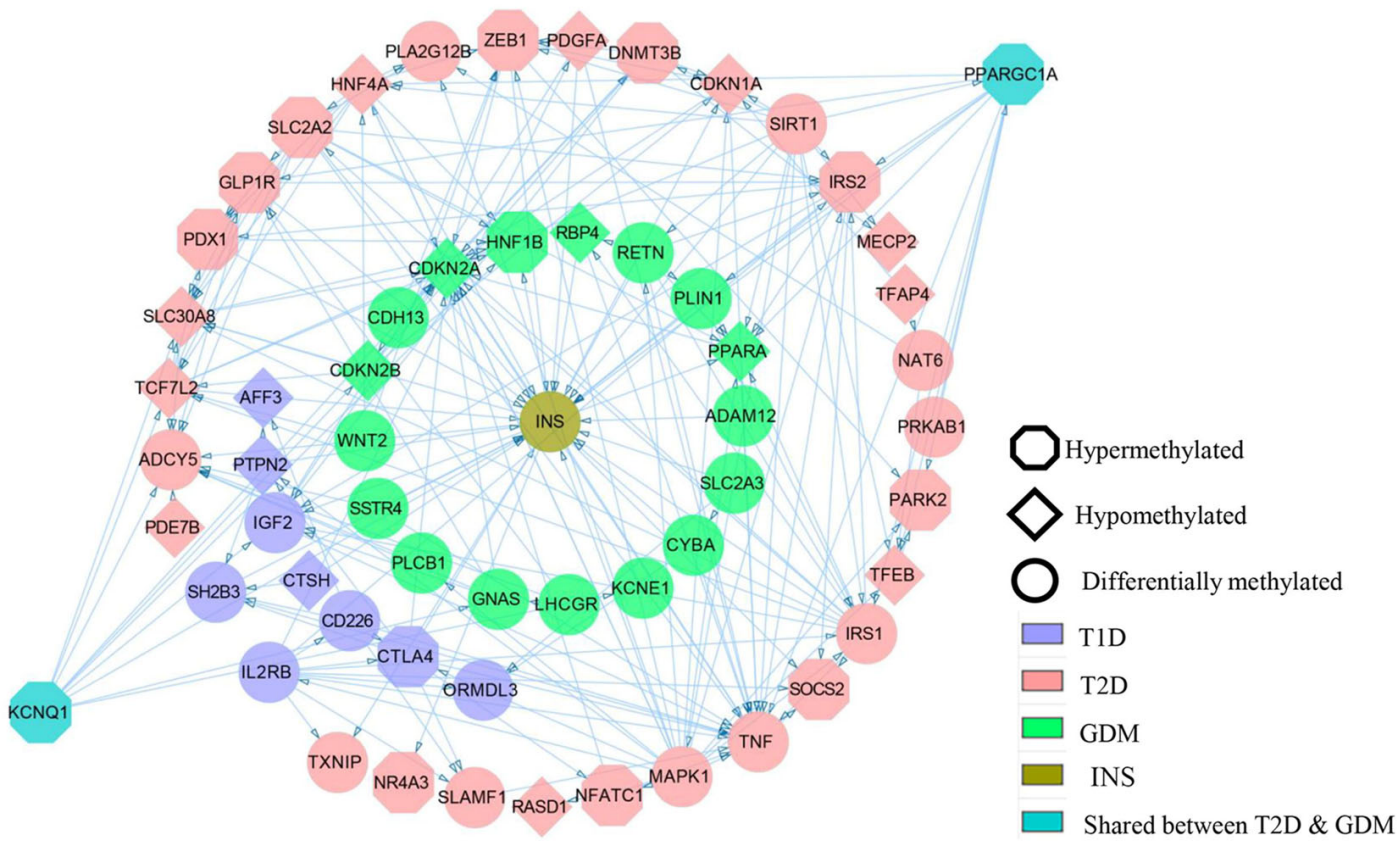
Gene network analysis of the gene promoters which are differentially methylated in T1D, T2D, and GDM. The arrows indicate the targets. Insulin is shown in the middle, whose expression is altered as a result of the change in the methylation level. Figure generated using String and Cytoscape. GDM, gestational diabetes mellitus [Color figure can be viewed at wileyonlinelibrary.com]

**Figure 4:**
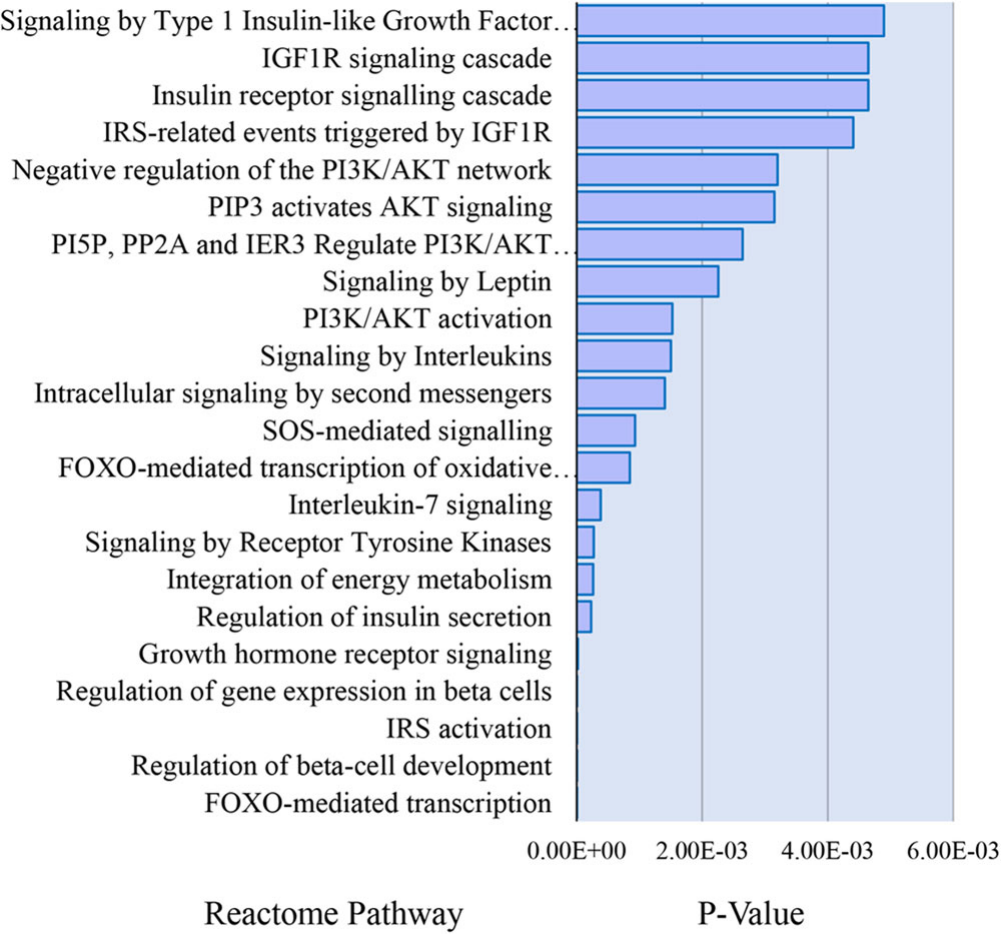
Pathway enrichment for the genes whose promoters are regulated by DNA methylation in the pancreas. The gene enrichment pathways for T1D, T2D, and GDM were generated using the web tool Enrichr, and the associated pathways were plotted using ggplot2 and R‐package. The picture show how Insulin and the associated signalling pathways are altered in T1D, T2D, and GDM in relation to the pancreas (*p* value <0.05). GDM, gestational diabetes mellitus [Color figure can be viewed at wileyonlinelibrary.com]

We have utilized all the genes which have been shown to have changes in expression and DNA methylation levels and were also linked to T1D, T2D and GDM and generated the gene network using STRING v11^139^ and visualized with the Cytoscape v3.8.0^140^ (Figure 3). This network shows, although most of these genes were analyzed individually, how they are connected to each other and finally affect INS. Further, along with the gene network, we have also generated the gene enrichment pathways for T1D, T2D and GDM using the web tool Enrichr and plotted the pathways using ggplot2 and R‐package^141^ (Figure 4). This shows how Insulin and the associated signalling pathways are altered through methylation in T1D, T2D and GDM with relation to the pancreas. The list of genes with changes in DNA methylation in diabetes is given in Table 2.

**Table 2:** List of genes with changes in DNA methylation in diabetes

Type 1 Diabetes				
Gene symbol	Methylation	Tissue	Function	References
AFF3	Hypomethylation	Blood	Scaffold for transcription elongation factor	^100^
CD226	Hypermethylation	Blood	Autoimmunity	^94^
CTLA4	Hypermethylation	Blood	Autoimmunity	^100^
CTSH	Hypomethylation	Blood	Antiapoptotic	^100^
HLA	Differentially methylated	β cell lines	Autoimmunity	^94^
HLADR	Differentially methylated	Blood/Mice islets	Autoimmunity	^102^
IGF2	Differentially methylated	Blood	Insulin signaling	^98^
IL2RB	Differentially methylated	β cell lines	Autoimmunity	^98^
Ins1	Differentially methylated	Mice beta cells	Metabolic homeostasis	^99^
Ins2	Differentially methylated	Mice beta cells	Metabolic homeostasis	^99^
ITGB3BP	Hypermethylation	Blood	Apoptosis/NFκB signaling	^100^
MEG3	Differentially methylated	CD14 + & CD4 + T cells	lncRNA	^98^
ORMDL3	Differentially methylated	CD14 + & CD4 + T cells	Innate immune signaling	^98^
PTPN2	Hypomethylation	Blood	Cell growth, differentiation and mitotic cycle	^100^
SH2B3	Differentially methylated	CD14 + & CD4 + T cells	Loss can cause thrombocythemia and erythrocytosis	^98^

**Type 2 diabetes**				
**Gene symbol**	**Methylation**	**Tissue**	**Function**	**References**
CDKN1A	Hypomethylation	Pancreatic islets	Impaired glucose‐stimulated insulin	^109^
CHRNA5	Differentially methylated	β‐cells	Glucose sensitivity	^108^
GLP1R	Hypermethylation	Pancreatic islets	Enhanced Insulin secretion	^112^
GLRA1	Hypermethylation	β‐cells	Glucose sensitivity	^108^
HNF4A	Hypomethylation	Liver	Pancreas development/fatty acid metabolism in hepatocytes	^88^, ^124^, ^126^
HOXC6	Hypomethylation	Skeletal Muscle	Sarcomere formation	^125^
IRS1	Differentially methylated	Skeletal Muscle	Insulin signaling	^113^
MAPK1	Differentially methylated	skeletal Muscle	Sarcomere formation	^125^
MECP2	Hypomethylation	Skeletal Muscle	Methylated CpG binding and repression	^129^
MYO18B	Differentially methylated	Skeletal Muscle	Sarcomere formation	^125^
NAT6	Hypermethylation	Skeletal Muscle	Hydrolysis of glycolipids	^129^
NFATC1	Hypomethylation	Muscle, mice β cells	Promotes β cell proliferation and mass	^129^
NR4A3	Hypermethylation	Pancreatic islets	β‐cell proliferation, regulation of myeloid differentiation	^114^
PARK2	Hypermethylation	Pancreatic islets	Mitophagy, maintenance of β‐cell function	^114^
PDE7B	Hypomethylation	Pancreatic islets	Impaired glucose‐stimulated insulin	^109^
PDGFA	Hypomethylation	Liver cells (IHHs)	Hyperinsulinemia and Insulin Resistance	^120^
PDX1	Hypermethylation	Pancreatic islets	Pancreatic development and β‐cells insulin expression	^108^, ^115^
PID1	Hypermethylation	Pancreatic islets	Glucose‐stimulated insulin secretion (GSIS)	^114^
PLA2G12B	Hypermethylated	Skeletal Muscle	Hydrolysis of glycolipids	^129^
PRKAB1	Differentially methylated	Skeletal Muscle	Sarcomere formation	^125^
RASD1	Hypomethylation	β‐cells	Glucose sensitivity	^108^
SEPT9	Hypomethylation	Pancreatic islets	Impaired glucose‐stimulated insulin	^109^
SIRT1	Hypermethylation	Skeletal Muscle	HDAC, positively regulates insulin secretion in Pancreatic β cells	^129^
SLC2A2	Hypermethylation	Pancreatic islets	glucose sensing, glucose transport and glucose homeostasis	^114^
SLC30A8	Hypermethylation	peripheral Blood	Insulin secretion	^111^
SLCO5A1	Hypermethylation	β‐cells	Glucose sensitivity	^108^
SOCS2	Hypermethylation	Pancreatic islets	Inflammation regulator, growth hormone (GH) signaling and pleiotropic E3 ligase	^114^
TCF7L2	Hypomethylation	Skeletal Muscle	Wnt signaling	^113^
TFAP4	Hypomethylation	Skeletal Muscle	Cell proliferation, EMT regulation	^129^
TFEB	Hypomethylation	Skeletal Muscle	lysosomal biogenesis and autophagy and lipid homeostasis	^129^
TNFα	Differentially methylated	Skeletal Muscle	Inflammation, Serine phosphorylation leading to impaired insulin signaling	^105^
TXNIP	Hypomethylation	Blood	insulin resistance and impaired islet cell function	^110^
VAC14	Hypomethylation	β‐cells	Glucose sensitivity	^108^
ZEB1	Hypermethylation	Skeletal Muscle	β cell differentiation	^129^
INS	Differentially methylated	β cell lines	Glucose homeostasis	^94^
PPARG	Hypomethylation	Skeletal Muscle	Fat and energy metabolism	^113^

**Gestational diabetes**				
**Gene symbol**	**Methylation**	**Tissue**	**Function**	References
ADAM12	Hypomethylation	Human placenta	Developmental pathways including Muscle and neural development	^133^
Cdh13	Differentially methylated	Mice Pancreas	Neuronal differentiation and development	^136^
CDKN2A/B	Hypomethylation	Rat Pancreatic Islets	Negative regulation of cell cycle progression	^135^
CYBA	Hypomethylation	Human Placentae	Metabolism	^131^
GLUT3	Hypomethylation	Human placenta	Glucose transportation	^132^
Hnf1b	Hypermethylation	Mice Pancreas	Transcription factor regulating multiple genes	^136^
KCNE1	Hypermethylation	Placenta	Regulator of potassium channel	^131^
Lhcgr	Differentially methylated	Mice Pancreas	GPCR receptor	^136^
Plcbr1	Differentially methylated	Human placenta	Signal transduction pathways	^136^
Plin1	Differentially methylated	Human placenta	Lipid storage in adipocytes	^138^
PPARα	Hypomethylation	Human placenta	Metabolism	^127^, ^132^
RBP4	Hypomethylation	Human placenta	Carrier of retinol	^133^
SSTR4	Differentially methylated	Human placenta	Hormone signaling	^138^
Wnt2	Differentially methylated	Mice Pancreas	Wnt signaling	^136^
Agap2	NA	Mice Pancreas	Netrin‐1 signalling and Development, HGF signalling pathway.	^136^
CCDC15	Hypomethylation	Human placenta		^133^
Fbp2	NA	Mice Pancreas	Gluconeogenesis	^136^
GSTM1	Hypomethylation	Human Placenta	Neutralization of reactive oxygen species	^131^
GSTM5	Hypomethylation	Human placenta	Neutralization of reactive oxygen species	^131^
Irx3	NA	Mice Pancreas	Neuronal development and vertebrate embryo patterning	^136^
NXN	Hypermethylation	Human Placenta	Redox homeostasis, (adipose) cell growth and differentiation	^131^
P2RX5	Hypomethylation	Human placenta	Purinoreceptor pathways of ATP	^133^
Resistin	Hypomethylation	Human placenta	Hormone linking obesity with diabetes	^132^
KCNQ1	Differentially methylated	Mice, Blood, Pancreas	Voltage‐gated potassium channel	^113^, ^115^, ^136^
PPARGC1A	Hypermethylation	Human Pancreatic islets	Adipocyte differentiation, Energy metabolism	^105^, ^106^, ^126^, ^128^

## 4 EPIGENETIC MODULATORS FOR THE TREATMENT OF DIABETES

The most prescribed drug for T2D is metformin. It reduces T2D by reducing intestinal glucose absorption, enhancing glucose uptake, decreasing plasma insulin levels at fasting, and increasing insulin sensitivity. It also inhibits gluconeogenesis. However, owing to side effects such as lactic acidosis, the search for better treatment options is still ongoing. Although epigenetic causes have been significantly attributed to the development and progression of diabetes, the field of epigenetic therapy for diabetes has not evolved much. This is probably because of the multifactorial cause of diabetes and the lack of personalized epigenetic information about patients. However, epigenetic therapy in combination with immunotherapy or other conventional therapeutic methods could be a good option for treating diabetes.

Genistein, an isoflavone of plant origin, has been found to be effective in treating obesity and insulin resistance by regulating the activities of enzymes for HGP and lipogenesis.^142^ Although the role of genistein as an epigenetic modulator is less explored, in androgen‐dependent prostate cancer (LNCaP) cells, it was shown to induce DNMT1 and HDAC1 expression.^143^ In kidney cells of mice, it has been shown to inhibit histone 3 deacetylation at promoters and normalize the promoter DNA hypermethylation.^144^ It also downregulates the expression of miR‐222 and promotes lipolysis.^145^ Genistein has undergone several clinical trials as a treatment against various types of cancers and also as an inducer of insulin sensitivity in patients with metabolic syndrome (ClinicalTrials.gov Identifier: NCT04105023). However, more studies are required to reveal its potential as an epigenetic modulator in diabetic patients.

Decitabine (5‐aza‐2′‐deoxycytidine; Aza) is an analogue of deoxycytidine, and when incorporated into the DNA, it inhibits DNA methylation. It has been approved by the Food and Drug Administration (FDA), USA, for the treatment of Chronic Myeloid Leukaemia (CML) and tested for its treatment potential against other types of cancers.^146^ Diabetes of any type eventually leads to apoptosis or dedifferentiation of pancreatic β‐cells, and therefore, regenerative therapy is one of the strategies commonly considered for its treatment. Treatment with decitabine, 5‐AZA‐2′‐deoxycytidine, a DNA methyltransferase inhibitor used in triatic myelodysplastic syndromes, has been found to be effective in inducing pancreatic β cell type features in human mesenchymal stem cells, including transcriptional activation of insulin, glucose transporter 2, glucokinase, and transcription factors MafA and NKX6.1 in vitro.^147^

NaB is a four‐carbon fatty acid that possesses general HDAC inhibitor activity.^148^ It has been proposed for the treatment of juvenile diabetes (especially T1D) based on the observation that it improves glucose homeostasis by improving β cells proliferation and function, decreasing β cells apoptosis, and modulating the p38/ERK MAPK pathway. Treatment with NaB results in significant improvement in plasma glucose levels, plasma insulin levels/expression, ameliorated diabetes‐induced histological alternations, and increased the expression of phosphorylated AKT.^149^ It also induces nuclear‐encoded mitochondrial genes for −1 nucleosome repositioning and causes muscle mitochondrial adaptations in skeletal muscles. Treatment with NaB eventually leads to improved β‐oxidation and a lean, insulin‐sensitive phenotype.^150^ NaB treatment in db/db mice has been shown to improve glycogen metabolism in the liver. It increased the levels of Gpr43, Glut2 and Sodium‐glucose cotransporter 1 (Sglt1) on the cell membrane, activated Gsk3 and inhibited Akt in liver cells.^151^ In T2D rat models generated using low dose STZ and HFD, treatment with NaB also reduces cholestenone and low‐density lipoprotein c (LDLc) and enhances insulin resistance and glucose tolerance. It improved diabetes‐induced histological alterations of islets and their functional damage.^152^ NaB treatment helps to downregulate ER stress proteins like phosphorylated Type 1 transmembrane ER‐resident protein kinase (PerkK), phosphorylated eukaryotic initiation factor (pelF2α), activator transcription factor (Atf) and CCAAT/Enhancer binding protein homologous protein (Chop).^152^ In HFD fed mice, NaB treatment prevented insulin resistance and preserved fasting blood glucose, fasting insulin, and insulin tolerance. Skeletal muscles showed elevated expression of peroxisome proliferator‐activated receptor‐gamma coactivator 1‐alpha (Pgc‐1α) and elevated activities of AMP kinase and p38.^153^ NaB treatment has been shown to improve STZ‐induced diabetes by downregulating NFκ‐B mediated inflammatory pathway through inhibiting Hmgb1.^154^ A clinical trial of NaB for treatment of diabetes and associated health conditions such as inflammation and albuminuria has been ongoing since 2019 (ClinicalTrials.gov Identifier: NCT04073927), and the results are awaited.

Subcutaneous injection of liraglutide, an analogue of glucagon‐like peptide (GLP‐1), is being used for treating T2D. Administration of it once a day helps to maintain both fasting and postprandial blood glucose at normal levels for 24 h, owing to its slow release and reduced renal filtration.^155^ It also improves the metabolic profile of obese T2D patients epigenetically by regulating *SIRT1* and downregulating the expression of the proinflammatory NF‐κB pathway.^156^ In Wistar rat models for catch‐up growth after nutrient deprivation, metabolic diseases set in due to the suppression of *Pdx*‐*1* resulting from the increased recruitment of the repressive chromatin modification H3K9me2 and reduced recruitment of activating chromatin modifications such as H3K4me3 and H3 acetylation at its promoter region. Treatment with liraglutide could reverse these epigenetic modifications and activate *Pdx‐1* expression enhancing β cell function.^157^ Many clinical trials have been conducted with liraglutide. One such study (ClinicalTrials.gov Identifier: NCT01299012), where the drug was subcutaneously administered daily at a rate of 0.6 mg, showed improved blood glucose control in T1D patients. Another study (ClinicalTrials.gov Identifier: NCT01392898) was conducted to understand the effect of liraglutide on insulin treatment‐associated weight gain in patients with T2D, and it showed that treatment with 1.8 mg of liraglutide improved glycemic control and reduced weight gain. A Phase 4 study (ClinicalTrials.gov Identifier: NCT03011008) focused on the use of liraglutide as an additional treatment to insulin in patients with autoimmune diabetes mellitus is still ongoing. A phase 2 study to understand the impact of liraglutide on glucose tolerance and the risk of T2D in women with previous pregnancy‐induced diabetes is ongoing (ClinicalTrials.gov Identifier: NCT01795248).

**Figure 5:**
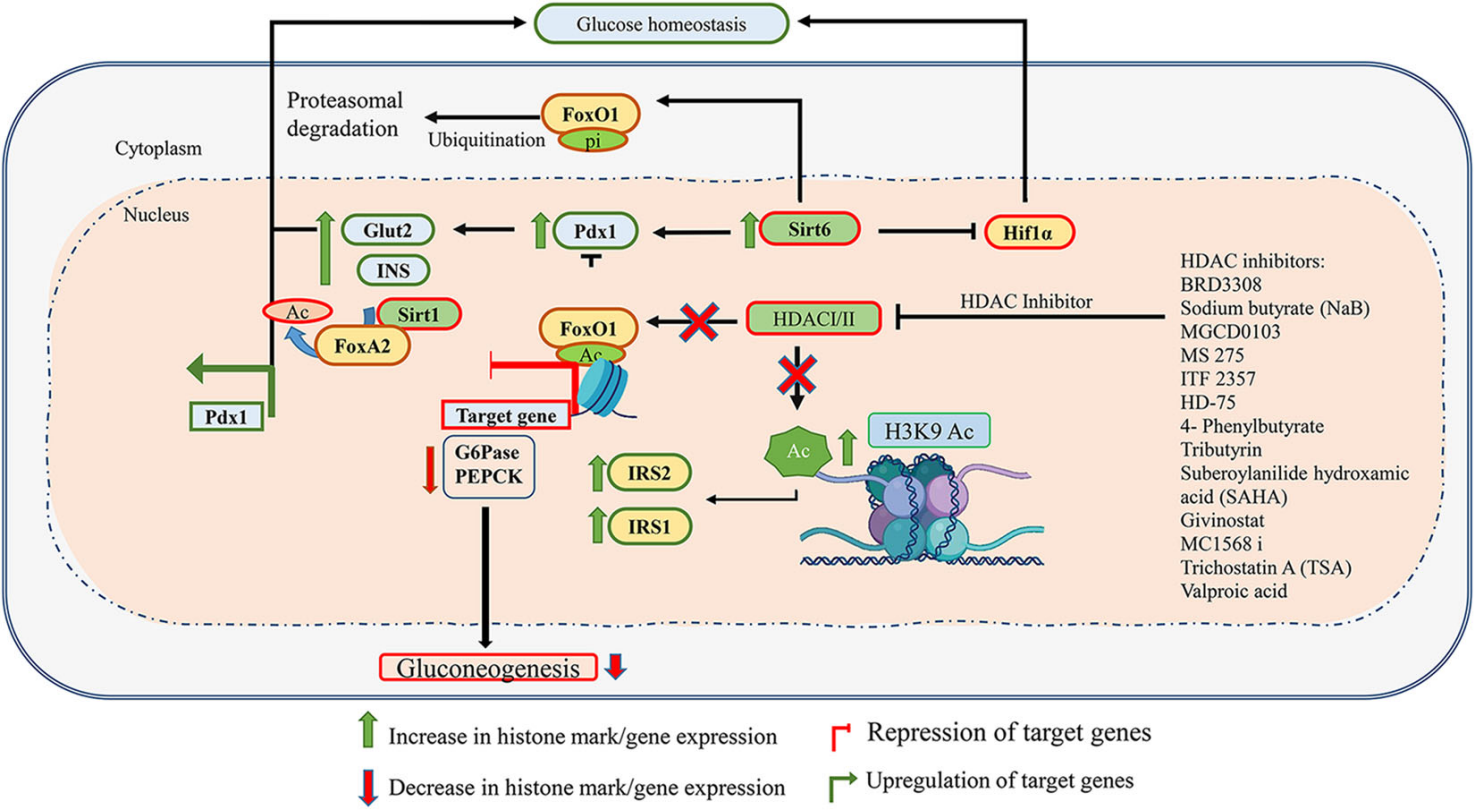
Schematic representation of the role of HDACs and HDAC inhibitors in glucose homeostasis. HDAC I/IIs catalyze the deacetylation of target histones and aid in the compaction of chromatin, which restricts transcription factors access to DNA, resulting in target gene repression. The inhibition of HDAC I/II maintains histone acetylation at the IRS2 promoter and promotes IRS2 expression. HDAC inhibition resulting in FoxO1 acetylation also maintains glucose homeostasis. HDAC III (e.g., SIRT1/SIRT2) activation promotes PDX1 expression through deacetylation of FoxA2, thereby maintaining β‐cell development and insulin secretion. Figure generated using BioRender. Green arrows show upregulation, and red arrows show downregulation. HDAC, histone deacetylase [Color figure can be viewed at wileyonlinelibrary.com]

The use of HDAC inhibitors also has the potential as therapeutic agents for diabetes as they promote β‐cell differentiation, proliferation, regeneration, and function and improve insulin resistance. The immunomodulatory action HDAC inhibitors such as suberoylanilide hydroxamic acid (SAHA) and Givinostat offers protection from developing T1D by preventing β‐cell destruction.^158^ Following treatment with SAHA, mesenchymal stromal cells were able to differentiate into insulin‐secreting cells.^147^ SAHA has already been approved by the FDA for its use in the treatment of cutaneous T cell lymphoma. Another potential agent, MC1568, a small molecular inhibitor of HDAC7, protects β‐cells from mitochondrial dysfunction and apoptosis and increases glucose‐stimulated insulin secretion in islets.^59^ Trichostatin A (TSA) and VPA are some of the other HDAC inhibitors under consideration for diabetes treatment.^53^ The generalized function of HDACs and the role of some of the HDAC inhibitors is shown in Figure 5.

## 5 CONCLUSION

Together, the studies presented substantiate the role of epigenetic regulatory mechanisms in normal development, differentiation, and the function of the pancreas and changes in them leading to the metabolic disease diabetes. DNA methyltransferases and histone‐modifying enzymes mediate gene expression, and through the regulatory interactions of transcription factors, they coordinate cell fate decisions and the differentiation of the pancreas. Genome‐wide studies have generated whole‐genome epigenomic maps that are good resources for understanding pancreatic development and differentiation and the contribution of epigenetics in the process. It also suggests how changes in their regulation can result in different diseases, including diabetes. Over the years, attempts have been made to establish methods by which damaged or destroyed β‐cells could be functionally replaced or compensated through the reprogramming of other types of cells to β‐cells, directed differentiation of stem cells to functional β‐cells, or stimulation of β‐cell proliferation to compensate for the loss.

Although previous studies have led to a better understanding of the development and differentiation of the pancreas and the transcription factors that are critical for each stage of pancreas specification as well as the chromatin states of the different cell populations during development and differentiation, which include stem cells, pancreatic progenitors, and different cell types of the islets, recent studies have only begun to assess whether chromatin regulators may be used as therapeutic targets for diabetes. Studies have shown that epigenetic reversion has the potential to improve cancer treatment, and thus, discovering epigenetic agents with the potential to change the epigenetic effect could be applied to treat diabetes and related pathologies. It is also becoming clearer it is the combination of DNA methylation and histone modifications together drive changes in regulatory mechanisms resulting in metabolic diseases such as diabetes. However, such studies are only becoming to be addressed.

Together, the fundamental studies and clinical observations of epigenetic regulatory mechanisms have provided new insights into the development and differentiation of the pancreas and how changes in them can contribute to diseases. These studies have also opened up new disease biomarkers and therapeutic targets that could promote the design of novel therapeutic strategies.

## CONFLICT OF INTERESTS

The authors declare that there are no conflict of interests.

## AUTHOR CONTRIBUTIONS

All authors contributed to the study design and data collection. **Suneesh Kaimala** and **Challagandla Anil Kumar**: wrote the initial version of the paper with contributions from **Bright Starling Emerald, Mohammed Z. Allouh**, and **Suraiya Anjum Ansari**. **Challagandla Anil Kumar**: generated the figures with help from **Bright Starling Emerald**. All authors reviewed, edited the manuscript and approved the final manuscript.

## Data Availability

There is no specific datasets generated as part of this review.
